# Marker events associated with adherence to HIV/AIDS treatment in a cohort study

**DOI:** 10.11606/s1518-8787.2023057004219

**Published:** 2023-03-29

**Authors:** Rafael Steffens Martins, Daniela Riva Knauth, Alvaro Vigo, Patrícia Fisch

**Affiliations:** I Universidade Federal do Rio Grande do Sul Faculdade de Medicina Programa de Pós-Graduação em Epidemiologia Porto Alegre RS Brasil Universidade Federal do Rio Grande do Sul. Faculdade de Medicina. Programa de Pós-Graduação em Epidemiologia. Porto Alegre, RS, Brasil; II Universidade Federal do Rio Grande do Sul Faculdade de Medicina Departamento de Medicina Social Porto Alegre RS Brasil Universidade Federal do Rio Grande do Sul. Faculdade de Medicina. Departamento de Medicina Social. Porto Alegre, RS, Brasil; III Universidade Federal do Rio Grande do Sul Instituto de Matemática e Estatística Departamento de Estatística Porto Alegre RS Brasil Universidade Federal do Rio Grande do Sul. Instituto de Matemática e Estatística. Departamento de Estatística. Porto Alegre, RS, Brasil

**Keywords:** Acquired Immunodeficiency Syndrome, drug therapy, Medication Adherence, Patient Dropouts, Risk Factors, Cohort Studies

## Abstract

**OBJECTIVE:**

To analyze how clinical and social events may impact adherence to antiretroviral treatment for HIV.

**METHODS:**

This is a historical cohort study with 528 patients who underwent treatment for HIV in a specialized care service in Alvorada, RS. A total of 3429 queries executed between the years 2004 and 2017 were analyzed. For each visit, data on treatment characteristics and the patients’ clinical picture were collected. Adherence, as measured by patients’ self-report, was the endpoint of the study. The logistic regression model via generalized estimating equations was used for estimating the associations.

**RESULTS:**

67.8% of the patients analyzed have up to 8 years of education and 24.8% have a history of crack and/or cocaine use. Among men, being asymptomatic [odds ratio (OR) = 1.43; 95%CI 1.05-1.93], having more than 8 years of education (OR= 2.32; 95%CI 1.27-4.23), and never having used crack (RC = 2.35; 95%CI 1.20-4.57) were associated with adherence. For women, being older than 24 years (CR = 1.82; 95%CI 1.09-3.02), never having used cocaine (CR = 2.54; 95%CI 1.32-4.88) and being pregnant (RC = 3.28; 95%CI 1.83-5.89) increased the odds of adherence.

**CONCLUSIONS:**

In addition to defined sociodemographic characteristics, one-off events that may occur in the trajectory of patients on long treatment, such as starting a new pregnancy and not having symptoms, can impact patients’ chances of treatment adherence.

## INTRODUCTION

Non-adherence to HIV antiretroviral therapy (ART) is a multifactorial phenomenon present in both rich and poor countries^1^. Successful treatment requires commitment to daily medication, subject to possible side effects. With correct adherence to ART, a reduction in viral load to undetectable levels is expected, providing benefits for the quality of life of patients and hindering the sexual transmission of HIV to other people^1 , 2^.

There are several studies investigating sociodemographic characteristics associated with adherence to antiretroviral treatment. The literature on the topic has shown, for example, that factors such as female gender, young age, white color/race, and high income and education are associated with greater chances of adherence to ART^1 - 3^. However, the complexity of adherence to long term treatment also occurs because of the possibility of changes in the frequency of adherence according to one-off events in the patients’ lives, for example, a new pregnancy^4 , 5^.

In Brazil, antiretroviral treatment for HIV has been universally available through the Brazilian Unified Health System (SUS) since 1996. Despite this, it is estimated that in 2018, only 66% of the 900,000 people living with HIV/AIDS (PLHIV) in the country were on treatment^6^. Analyzing patient adherence to ART, of the 637,000 patients who recorded at least one drug dispensation in 2018, approximately 17% had less than 80% adherence to antiretroviral drugs and 8% were classified as dropouts^6^. Given the context presented, this study seeks to understand how clinical and social events that present themselves in the trajectory of individuals can impact adherence to antiretroviral treatment for HIV.

## METHODS

The study data come from a historical cohort, called the Alvorada Cohort^7^, with PLWHA in the municipality of Alvorada, located in the Metropolitan Region of Porto Alegre. The cohort has information from 1093 patients who underwent HIV treatment at the only Specialized Care Service (SAE) in the study municipality. The data were systematized by collecting information from the consultations available in the SAE medical records.

The units of analysis for the study were the patients’ consultations. Initially, the cohort contained 10361 queries. For this work, we selected the consultations whose patients: (1) had prescribed medication for HIV treatment; (2) were 18 years or older; and (3) had attended the consultation. Following these criteria, 2177 consultations of patients who had no prescription for the drugs, 326 consultations of patients under 18 years of age, and 1770 consultations in which the patient did not show up at the SAE on the scheduled date were excluded. Then, exclusively those with information on ART adherence, clinical presentation, opportunistic infection, and hospitalization were selected. With this, the final sample of the study was 3,429 consultations performed between 2004 and 2017.

The endpoint of the study was patient adherence to ART. It was measured from the patients’ self-report at each visit, in which the health professional in charge would ask the patient about the use or non-use of the prescribed medications. In the data collection, adherence was classified as “regular adherence”, “irregular adherence”, or “non-adherent”. For this study, the variable was recoded into two categories: “in compliance”, when the patient is taking the medication regularly, or “in poor compliance”, when the patient is in irregular or non-adherent compliance.

Also measured in all consultations, the variables used to characterize the treatment and clinical picture of patients were: clinical presentation (symptomatic/asymptomatic), infection by opportunistic disease (yes/no), hospital admission (yes/no) and pregnancy (yes/no), in the case of women. The variable “hospital admission” considers whether the patient was admitted exclusively because of HIV complications.

Sociodemographic and behavioral information was collected only once at the time of patients’ entry to the SAE. They are: sex (male/female), education (by ranges of years studied), age (in years), marital status (single/dating/married or living together), HIV exposure category (heterosexual/homosexual/drug addiction/vertical transmission), alcohol abuse (yes/no), cocaine use (yes/no), and crack use (yes/no).

For the variable “alcohol abuse”, only the cases in which the individual had experienced alcoholism at some point in his or her life were considered. Cases in which the person reports occasional alcohol use were not considered. For the variables “crack use” and “cocaine use”, the cases in which the individuals reported using or having already used the drugs once in their lives were considered.

From factors such as “education” and “age” variables were created for analysis based on specific categories and values. For education, we created the dichotomous variable “high school”, whose categories represent: (1) having up to 8 years of study; and (2) having more than 8 years of study (which is equivalent to starting high school). For age, two age categories were created: (1) people aged 18 to 24, considered young; and (2) people over 24. The distinction was made taking into consideration the various studies on the topic that indicate that young people have lower chances of adhering to ART^1 , 3 , 8 , 9^.

The sociodemographic and behavioral characteristics of the patients were presented separately for men and women, as well as the frequencies of events (such as regular adherence, hospital admission, opportunistic infection) recorded throughout the follow-up. Comparisons of means were performed using *Student* ’s t-test and comparisons of proportions using Pearson’s chi-square-based test or *Fisher* ’s exact test.

The logistic regression model was used to assess the associations between clinical, sociodemographic, and behavioral factors and adherence to ART. To account for correlations between different visits to the same patient, generalized estimating equations (GEE) approach was used. As study factors, we analyzed: clinical presentation, hospitalization, opportunistic infection, and pregnancy (in the case of female patients). Sociodemographic and behavioral variables (young age, high school, alcohol, cocaine and crack abuse) were used as controls in the models.

The models were stratified by sex because of the presence of the variable “pregnancy”, included only in the analysis of women. For both genders, three models were constructed aiming to estimate the odds ratios (OR) of adherence to antiretroviral treatment. The first model considers only the exposures under study; the second includes sociodemographic control variables; and the third adds drug use variables.

The analyses were performed on the Statistical Package for the Social Sciences (SPSS) software, version 18, using the 5% significance level. The project was approved by the Research Ethics Committee of Universidade Federal do Rio Grande do Sul (CEP/UFRGS) and the researchers signed a term of commitment for the use of data.

## RESULTS

The sociodemographic and behavioral characteristics of the patients are presented in Table 1 . Of the 528 patients, 270 (51.1%) are male and 258 (48.9%) are female, with a median age of 37 years (IQR = 30-46) and 35 years (IQR = 27-44), respectively.

**Table 1:** Sociodemographic and behavioral characteristics of patients, by sex - median (IQR) or n (%).

Variable	Men (n = 270)	Women (n = 258)	p
Age - Median (IQR)	37 (30–46)	35 (27–44)	0.073^a^
Education (years)			
≤ 4	39 (14.4%)	42 (16.3%)	0.411^b^
5–8	137 (50.8%)	140 (54.3%)	
≥ 8	94 (34.8%)	76 (29.4%)	
Marital status			0.367^b^
Single	139 (51.9%)	116 (45.7%)	
Married/Living together	78 (29.1%)	83 (32.7%)	
Dating	51 (19.0%)	55 (21.7%)	
HIV exposure category			< 0.001^c^
Heterosexual	105 (38.9%)	219 (84.9%)	
Homosexual	48 (17.8%)	2 (0.8%)	
Injecting drug use	10 (3.7%)	0 (0%)	
Vertical transmission	0 (0%)	0 (0%)	
Ignored	107 (39.6%)	37 (14.3%)	
History of alcohol abuse?			< 0.001^b^
Yes	88 (32.6%)	32 (12.4%)	
No	182 (67.4%)	226 (87.6%)	
History of cocaine use?			0.003^b^
Yes	61 (22.6%)	33 (12.8%)	
No	209 (77.4%)	225 (87.2%)	
History of crack use?			0.167^b^
Yes	39 (14.4%)	27 (10.5%)	
No	231 (85.6%)	231 (89.5%)	

^a^Student’s t-test.

^b ^Test for equality of proportions based on Pearson’s chi-square-based statistic.

^c ^Fisher’s exact test.

Analyzing the use of licit and illicit drugs, the history of use for men is higher than for women: 32.6% of men and 12.4% of women have a history of alcohol abuse (p < 0.001); 22.6% of men and 12.8% of women have a history of cocaine use (p = 0.003), and 14.4% of men have a history of crack use, while in women the proportion is 10.5% (p = 0.167).

The number of records analyzed lacking information regarding the category of HIV exposure of the patients was significant: 39.6% among men and 14.3% among women. About 84.9% of the women in the sample acquired HIV through heterosexual intercourse - which represents almost all of the female patients with available information. In men, besides the large proportion of medical records without information, 38.9% of patients reported infection through heterosexual intercourse, 17.8% through homosexual intercourse and 3.7% due to injecting drug use (p < 0.001).

As for marital status, the proportions were similar between men and women: 51.9% of men and 45.7% of women were single, while 29.1% of men and 32.7% of women were married or living together with their partner. The education variable shows that most participants in the sample have 5 to 8 years of schooling, and that the proportion of people with up to 4 years of schooling was 14.4% for men and 16.3% for women. The variables education and marital status showed no statistically significant difference between the groups.

Table 2 describes the patient visits in relation to the occurrence of adherence failure, clinical presentation, hospital admission, opportunistic infections, and pregnancy (n = 3,429). Regarding adherence to ART, men reported adherence in 86.1% of the consultations, and women in 83.9%. The clinical presentation of the patients was also similar between the two groups: in 82.4% of the men’s visits and 81.5% of the women’s visits no clinical symptoms were reported. Men and women experienced hospital admissions for HIV-related complications in 2.2% and 1.9% of visits, respectively. Opportunistic infections occurred in 10 visits (0.6%) for men, and 4 (0.2%) visits for women. No statistically significant differences were found between the consultations of the two groups. Finally, female patients were found to be pregnant in 9.3% of all consultations.

**Table 2:** Factors associated with the number of consultations in men and women - n (%).

Factor	Number of consultations		p^a^

	Men (n = 1.663)	Women (n = 1.766)	
Have you been in regular adherence since the previous appointment?			0.285
Yes	1.432 (86.1%)	1.482 (83.9%)	
No	231 (13.9%)	284 (16.1%)	
Clinical presentation in consultation			0.534
Symptomatic	293 (17.6%)	327 (18.5%)	
Asymptomatic	1370 (82.4%)	1.439 (81.5%)	
Occurrence of hospitalization?			0.773
Yes	36 (2.2%)	33 (1.9%)	
No	1627 (97.8%)	1.733 (98.1%)	
Occurrence of opportunistic infection?			0.316
Yes	10 (0.6%)	4 (0.2%)	
No	1653 (99.4%)	1762 (99.8%)	
Pregnant during the consultation?			
Yes	-	165 (9.3%)	
No	-	1601 (90.7%)	

^a ^Estimated using the logistic regression model via generalized estimating equations.

Table 3 presents odds ratio estimates of ART adherence for men. In model 1, there were increased odds of adherence to antiretroviral therapy in visits in which patients were asymptomatic (CR= 1.46; 95%CI 1.09-1.97) and were not hospitalized for HIV complications (CR = 1.43; 95%CI 1.06-1.92). The variable “opportunistic infection” was not statistically significant.

**Table 3:** Odds ratio estimates of adherence to antiretroviral therapy in men.

Factors	Odds Ratio (95%CI)		

	Model 1	Model 2	Model 3
Clinical presentation			
Symptomatic	1	1	1
Asymptomatic	1.46^a^ (1.09–1.97)	1.43^a^ (1.06–1.92)	1.43^a^ (1.05–1.93)
Hospital admissions			
Yes	1	1	1
No	1.86^a^ (1.00–3.46)	1.85 (0.99–3.45)	1.82 (0.96–3.44)
Opportunistic infection			
No	1	1	1
Yes	0.69 (0.19–2.50)	0.70 (0.19–2.51)	0.65 (0.16–2.54)
Age (years)			
8–24	-	1	1
≥ 24	-	0.68 (0.27–1.72)	0.62 (0.24–1.58)
Education (years)			
< 8	-	1	1
> 8	-	2.32^a^ (1.27-4.23)	1.82 (0.97–3.41)
History of alcohol abuse			
Yes	-	-	1
No	-	-	1.42 (0.81–2.47)
History of cocaine use			
Yes	-	-	1
No	-	-	0.85 (0.45–1.60)
History of crack use			
Yes	-	-	1
No	-	-	2.35^a^ (1.20-4.57)

^a^p < 0,05.

In model 2, with sociodemographic variables, being asymptomatic (CR = 1.43; 95%CI 1.06-1.92) and no record of hospitalization (CR= 1.85; 95%CI 0.99-3.45) there were still increased odds of adherence for men, although the second variable lost statistical significance. In this model, patients with more than 8 years of education have increased odds of adherence (CR = 2.32; 95% CI 1.27-4.23). In model 3, only clinical presentation remained significant, increasing the odds of adherence by about 43% in the absence of symptoms (CR = 1.43; 95% CI 1.05-1.93). Regarding drug use, never having used crack appears as a factor associated with adherence (CR = 2.35; 95% CI 1.20-4.57).

Table 4 presents the three models with only the women’s queries. In the former, being pregnant more than doubles the odds of adhering to treatment (CR = 2.71; 95% CI 1.49-4.91), while the absence of symptoms increases the odds, although without statistical significance.

**Table 4:** Odds ratio estimates of adherence to antiretroviral therapy in women.

Factors	Odds Ratio (95%CI)		

	Model 1	Model 2	Model 3
Clinical presentation			
Symptomatic	1	1	1
Asymptomatic	1.18 (0.89–1.57)	1.20 (0.90–1.60)	1.22 (0.91–1.64)
Hospital admissions			
Yes	1	1	1
No	0.84 (0.37–1.93)	0.83 (0.36–1.91)	0.83 (0.36–1.92)
Opportunistic infection			
No	1	1	1
Yes	2.15 (0.12–38.15)	2.19 (0.10–45.88)	2.23 (0.17–28.62)
Pregnancy			
No	1	1	1
Yes	2.71^a^ (1.49-4.91)	3.07^a^ (1.72-5.48)	3.28^a^ (1.83-5.89)
Age (years)			
18–24	-	1	1
≥ 24	-	1.79^a^ (1.08-2.96)	1.82^a^ (1.09-3.02)
Education (years)			
< 8	-	1	1
> 8	-	1.29 (0.78–2.13)	1.22 (0.74–2.03)
History of alcohol abuse			
Yes	-	-	1
No	-	-	0.63 (0.29–1.34)
History of cocaine use			
Yes	-	-	1
No	-	-	2.54^a^ (1.32-4.88)
History of crack use			
Yes	-	-	1
No	-	-	1.06 (0.50–2.22)

^a^p < 0.05.

Adding the sociodemographic variables, model 2 remains with similar results to the previous one, in which pregnancy (CR = 3.07; 95% CI 1.72-5.48) increases the odds of adherence by almost three times. The variable age also shows an association, increasing the chances in people over 24 years old (CR = 1.79; 95% CI 1.08-2.96). Finally, in model 3, pregnant patients at the consultation have more than a threefold increase in the odds of adherence (CR = 3.286; 95%CI 1.832-5.895), while not being young (CR = 1.82; 95%CI 1.09-3.02), and not using cocaine (CR = 2.54; 95%CI 1.32-4.88) significantly increase the odds of adhering.

## DISCUSSION

Several studies point to sociodemographic and behavioral factors that may influence patients’ chances of adhering to ART. In many of these papers, adherence is treated in a “definitive” and not very flexible way: the individual is either adherent or not^1 - 3 , 10^. However, it is not only sociodemographic characteristics that can impact treatment adherence, since one-off events that occur throughout patients’ lives can also influence these chances^11^. These events include: the occurrence of a pregnancy; hospitalization; infection by an opportunistic disease; and clinical presentation.

In this study, pregnant women have up to a 3.28 greater chance of adhering to ART compared to women who are not pregnant. The literature points out that pregnancy can impact adherence because: (1) many women start treatment together with prenatal care, in which part of them discovers their HIV serology; (2) there is an attention of policies for this population, focused mainly on maternal-infant care; (3) there are feelings and fears, on the part of the pregnant woman, of transmitting HIV to her child^5 , 12^.

In fact, several incentives are used in prenatal care to stimulate adherence to ART, for example: greater attention from health teams to pregnant women; strict monitoring of patients to avoid vertical transmission of HIV; active search for pregnant women who do not attend health services, etc. Thus, it is expected that these incentives stimulate an increase in adherence during pregnancy. In another analysis of data from the Alvorada cohort, it was evidenced that pregnant women take less time to reach an undetectable HIV viral load when compared to non-pregnant patients^7^. On the other hand, several studies show that after delivery, the chances of treatment failure or even abandonment increase^5 , 13 , 14^.

This indicates that pregnancy can be a one-time event that positively impacts treatment adherence precisely because of the way this population is monitored during prenatal care, when they receive more attention from health policies and greater encouragement to build the link with health services. After pregnancy ends, however, these stimuli are weakened.

The results of this study contribute to the understanding of the effect of pregnancy on the lives of women living with HIV and are relevant especially when analyzing the incidence of HIV diagnoses in pregnant women in the country and in the metropolitan region where Alvorada is located. In Brazil, the HIV detection rate in pregnant women has grown 38.1% in the last 10 years. Among Brazilian capitals, Porto Alegre recorded the highest HIV detection rate in pregnant women in the country in 2018^15^.

These analyses suggest that the men in the sample seek to maintain correct adherence even when they do not have HIV complications, unlike other studies of chronic diseases that show that the absence of symptoms makes patients stop taking medication due to lack of understanding of the course of the disease or because they believe that it is no longer necessary to take the drugs^22 , 23^.

However, this result can also be analyzed through the direct relationship between adherence to ART and the expected symptomatic improvement of patients, since the effectiveness of treatment and the absence of symptoms depend directly on adherence. Studies show that to ensure viral suppression and a significant reduction in the risk of death, patients must correctly take at least 95% of the prescribed medication^20 , 21^.

Analyzing the relationship between clinical presentation and adherence in the literature, papers show that the absence of symptoms can be a reason to adhere to medication, although symptomatic improvement is, in some cases, associated with treatment abandonment, since the patient may judge that they no longer needs the medication^1 , 11^. On the other hand, presenting symptoms can also make the patient choose to abandon treatment because they believe that the worsening symptoms are related to the medications^16^.

This kind of “self-management” of the medications ^17^ and daily choice to use them or not is common in long treatments such as chronic diseases. After the development of antiretroviral therapy, AIDS underwent a transformation on its aspect from acute to chronic disease. That is, if before the disease was associated with the certainty of death, today it is possible to live with HIV normally ^18 , 19^. As with other chronic diseases, regular treatment compliance may not occur even with symptoms of the disease. It is also hindered because, in some cases, patients do not see treatment as being a long and continuous process, which is a characteristic of chronic diseases^11^.

Among the sociodemographic and behavioral variables, an association was found between age and adherence to ART, where being older than 24 years increases the chance of adhering to ART by up to 82% among women. There are several studies investigating factors associated with adherence to ART, pointing out that young people are less likely to adhere to both antiretroviral treatment and other chronic diseases^1 , 3 , 8 , 9 , 22^. Besides the difficulties in adhering, recent data have shown that young people are one of the groups that have had the highest increase in HIV detection rates in recent years, highlighting the vulnerability of them in the current scenario of the epidemic. Among young people aged 20 to 24, the detection rate has increased 94.6% in the last decade^15^.

The use of drugs such as crack and cocaine is also mentioned in the literature several times as a factor that has a major impact on ART^1 - 3^. An extensive review of the literature developed on the subject shows that several cases are reported in which substance use appears associated with HIV complications, since they reduce the chances of patient adherence to antiretroviral treatment^24^. In this study, it was found that having no history of crack and cocaine use significantly increases the chances of adherence. Other research has shown that drug use has a negative effect on the chances of adhering, both in men and women and in rich or poor countries^25 , 26^. Studies and reviews involving the use of ART also show that people who use drugs have greater difficulties in adherence, even with the presence of symptoms and clinical pictures evolving to AIDS^1 , 2 , 24^.

It should be noted that, unlike other studies, our sample was composed of consultations, which may be related to the high adherence found in the study (86.1% in men’s consultations and 83.9% in women’s consultations). It is expected that patients who attend appointments are more likely to follow treatment, while people with irregular adherence tend to miss more appointments or even discontinue treatment, making the proportion of appointments with adhering patients in the sample naturally increase. In addition, 1,170 consultations (17.1% of all consultations in the cohort) were excluded from the analyses because the patients did not attend. Finally, another factor that may be impacting this high proportion of adherence consultations is the very way in which the variable was measured. In some cases, patients’ self-report may be overestimated due to concern about not disappointing the healthcare professionals providing the care^11^.

The study sample includes a socially vulnerable population. More than half of the patients have up to eight years of education, which is equivalent to elementary school, and about a quarter have a history of crack and/or cocaine use. Alvorada is located in the Metropolitan Region of Porto Alegre and is the 2nd municipality with the lowest GDP per capita in Rio Grande do Sul^28^. In addition, the municipality scores lower than the national average in education and development statistics^27 , 28^.

This study used secondary data, which limits the data analysis to only information recorded in the medical records. In this sense, other characteristics associated with adherence discussed in the literature were not explored. In addition, among the variables selected for the analyses, in many queries the information was not found, significantly reducing the number of units of analysis in the sample. It is also important to reiterate that the way adherence was measured may influence the results, since patients may refer greater adherence to health professionals in order not to disappoint them^11^.

On the other hand, the great contribution of the study is to look at adherence in the patients’ trajectory. This perspective allowed us to demonstrate how some specific events that occur in the trajectory of patients in situations of vulnerability can impact treatment adherence. The influence of pregnancy on adherence suggests that prenatal care is important for the treatment of pregnant women living with HIV. However, just like pregnancy, the prenatal period is transient, and does not include all women.

In this sense, new strategies that stimulate permanent adherence deserve to be thought of to guarantee therapeutic success. Especially in the case of women, it is crucial to change strategies and implement public policies that support women also after pregnancy, when the chance of adherence is reduced. In addition, measures to strengthen the retention of postpartum women in health services (such as home visits, adherence consultations, and effective linkages with primary care) need to be encouraged to control the risk of vertical transmission through breastfeeding, often neglected by health policies.

The results of the paper point out that a good clinical presentation increases the chances of adherence to ART for men in the sample. In this sense, our data indicate the importance of integrating HIV prevention and care strategies, since patients with good compliance tend to have undetectable viral load, significantly decreasing the chances of virus transmission.
