# Supplementary material for: Marker events associated with adherence to HIV/AIDS treatment in a cohort study
Source: Rev Saude Publica. 2023 Mar 29;57:20. doi: 10.11606/s1518-8787.2023057004219 (PMC10118423; doi:10.11606/s1518-8787.2023057004219)
Supplement: Translation

# Translation: Eventos marcadores associados à adesão ao tratamento para HIV/aids em um estudo de coorte

**Authors:** Rafael Steffens Martins, Daniela Riva Knauth, Alvaro Vigo, Patrícia Fisch

**Affiliations:** I Brasil Universidade Federal do Rio Grande do Sul. Faculdade de Medicina. Programa de Pós-Graduação em Epidemiologia. Porto Alegre, RS, Brasil; II Brasil Universidade Federal do Rio Grande do Sul. Faculdade de Medicina. Departamento de Medicina Social. Porto Alegre, RS, Brasil; III Brasil Universidade Federal do Rio Grande do Sul. Instituto de Matemática e Estatística. Departamento de Estatística. Porto Alegre, RS, Brasil

**Keywords:** Síndrome de Imunodeficiência Adquirida, tratamento farmacológico, Adesão à Medicação, Pacientes Desistentes do Tratamento, Fatores de Risco, Estudos de Coortes

## Abstract

**OBJETIVO:**

Analisar como eventos clínicos e sociais podem impactar na adesão ao tratamento antirretroviral para o HIV.

**MÉTODOS:**

Trata-se de um estudo de coorte histórica com 528 pacientes que realizaram o tratamento para o HIV em um serviço de assistência especializada em Alvorada, RS. Foram analisadas 3429 consultas executadas entre os anos de 2004 e 2017. Para cada consulta, foram coletados dados de características do tratamento e do quadro clínico dos pacientes. A adesão, aferida pelo autorrelato dos pacientes, foi o desfecho do estudo. O modelo de regressão logística via equações de estimação generalizadas foi utilizado para estimação das associações.

**RESULTADOS:**

67,8% dos pacientes analisados possuem até 8 anos de estudos e 24,8% têm histórico de uso de crack e/ou cocaína. Entre os homens, estar assintomático [razão de chances (RC) = 1,43; IC95% 1,05–1,93], possuir mais de 8 anos de estudo (RC = 2,32; IC95% 1,27–4,23) e nunca ter usado crack (RC = 2,35; IC95% 1,20–4,57) estiveram associados à adesão. Para as mulheres, possuir mais de 24 anos (RC = 1,82; IC95% 1,09–3,02), nunca ter usado cocaína (RC = 2,54; IC95% 1,32–4,88) e estar em gestação (RC = 3,28; IC95% 1,83–5,89) aumentaram as chances de adesão.

**CONCLUSÕES:**

Além de características sociodemográficas definidas, eventos pontuais que podem ocorrer na trajetória de pacientes em tratamentos longos, como início de uma nova gestação e não apresentar sintomas, podem impactar nas chances de adesão dos pacientes ao tratamento.

## INTRODUÇÃO

A não adesão à terapia antirretroviral para o HIV (TARV) é um fenômeno multifatorial presente tanto em países ricos como em pobres^1^. O sucesso do tratamento exige comprometimento à medicação diária, sujeita a possíveis efeitos colaterais. Em correta adesão ao TARV, espera-se uma redução na carga viral a níveis indetectáveis, proporcionando, além dos benefícios para a qualidade de vida dos pacientes, uma redução no risco de transmissão sexual do HIV para outras pessoas^1 , 2^.

Diversos são os estudos que investigam características sociodemográficas associadas à adesão ao tratamento antirretroviral. A literatura sobre o tema tem mostrado, por exemplo, que fatores como gênero feminino, idade jovem, cor/raça branca, e alta renda e escolaridade estão associados a maiores chances na adesão ao TARV^1^. No entanto, a complexidade da aderência a tratamentos longos também ocorre devido à possibilidade de mudanças na frequência da adesão de acordo com eventos pontuais na vida dos pacientes, por exemplo, uma nova gestação^4 , 5^.

No Brasil, o tratamento antirretroviral para o HIV está disponível pelo Sistema Único de Saúde (SUS), de forma universal, desde 1996. Apesar disso, estima-se que, em 2018, somente 66% das 900 mil pessoas vivendo com HIV/aids (PVHA) no país estavam em tratamento^6^. Analisando a aderência dos pacientes ao TARV, dos 637 mil pacientes que registraram pelo menos uma dispensação de medicamento em 2018, aproximadamente 17% apresentaram adesão inferior a 80% dos medicamentos antirretrovirais e 8% foram classificados como em abandono^6^. Frente ao contexto apresentado, este estudo procura compreender como eventos clínicos e sociais que se apresentam na trajetória dos indivíduos podem impactar na adesão ao tratamento antirretroviral para o HIV.

## MÉTODOS

Os dados do estudo são provenientes de uma coorte histórica, chamada Coorte de Alvorada^7^, com PVHA no município de Alvorada, localizado na Região Metropolitana de Porto Alegre. A coorte possui informações de 1093 pacientes que realizaram tratamento para o HIV no único Serviço de Assistência Especializada (SAE) do município do estudo. Os dados foram sistematizados através de uma coleta realizada a partir das informações das consultas disponíveis nos prontuários do SAE.

As unidades de análises do estudo foram as consultas dos pacientes. Inicialmente, a coorte continha 10361 consultas. Para esse trabalho, foram selecionadas as consultas cujos pacientes: (1) possuíam medicação prescrita para o tratamento do HIV; (2) tinham 18 anos ou mais; e (3) haviam comparecido à consulta. Seguindo estes critérios, foram excluídas 2177 consultas de pacientes que não possuíam prescrição aos medicamentos, 326 consultas de pacientes com menos de 18 anos de idade, e 1770 consultas em que o paciente não compareceu ao SAE na data agendada. Em seguida, foram selecionadas somente aquelas que obrigatoriamente possuíam informações de adesão ao TARV, apresentação clínica, registro de infecção oportunista e hospitalização. Com isso, a amostra final do estudo foi de 3.429 consultas realizadas entre 2004 e 2017.

O desfecho do estudo foi a adesão dos pacientes ao TARV. Ela foi aferida a partir do autorrelato dos pacientes em cada consulta, em que o profissional de saúde responsável questionava o paciente a respeito da utilização ou não dos medicamentos prescritos. Na coleta de dados, a adesão foi classificada como “adesão regular”, “irregular” ou “não aderente”. Para este estudo, a variável foi recodificada em duas categorias: “em adesão”, quando o paciente está tomando a medicação regularmente, ou “em má adesão”, quando o paciente está em adesão irregular ou não aderente.

Também aferidas em todas as consultas, as variáveis utilizadas para caracterizar o tratamento e quadro clínico dos pacientes foram: apresentação clínica (sintomático/assintomático), infecção por doença oportunista (sim/não), internação hospitalar (sim/não) e gestação (sim/não), no caso das mulheres. A variável “internação hospitalar” considera se o paciente foi internado exclusivamente por conta de complicações em função do HIV.

Informações sociodemográficas e comportamentais foram coletadas apenas uma vez no momento de entrada dos pacientes ao SAE. São elas: sexo (homem/mulher), escolaridade (por faixas de anos estudados), idade (em anos), situação conjugal (solteiro/namorando/casado ou morando junto), categoria de exposição ao HIV (heterossexual/homossexual/drogadição/transmissão vertical), abuso de álcool (sim/não), uso de cocaína (sim/não) e uso de crack (sim/não).

Para a variável “abuso de álcool”, foram considerados apenas os casos em que o indivíduo passou por um quadro de etilismo em algum momento da vida. Não foram considerados casos em que a pessoa relata uso eventual de álcool. Já para as variáveis “uso de crack” e “uso de cocaína”, foram considerados os casos em que os indivíduos relataram usar ou já terem feito uso das drogas uma vez na vida.

A partir de fatores, como “escolaridade” e “idade”, foram criadas variáveis para análises com base em categorias e valores específicos. Para a escolaridade, criou-se a variável dicotômica “ensino médio”, cujas categorias representam: (1) possuir até 8 anos de estudo; e (2) possuir mais de 8 anos de estudo (o que equivale ao início do ensino médio). Para a idade, foram criadas duas categorias de faixa etária: (1) pessoas de 18 a 24 anos, consideradas jovens; e (2) pessoas acima de 24 anos. A distinção foi realizada levando em consideração os diversos trabalhos sobre o tema que indicam que jovens possuem menores chances de adesão ao TARV^1 , 3 , 8 , 9^.

As características sociodemográficas e comportamentais dos pacientes foram apresentadas separadamente para homens e mulheres, assim como as frequências de eventos (como adesão regular, internação hospitalar, infecção oportunista) registrados ao longo do seguimento. Comparações de médias foram realizadas por meio do teste t de *Student* e comparações de proporções por meio do teste baseado na estatística qui-quadrado de Pearson ou teste exato de *Fisher* .

O modelo de regressão logística foi utilizado para avaliar as associações entre fatores clínicos, sociodemográficos e comportamentais e a adesão ao TARV. Para considerar as correlações entre diferentes consultas em um mesmo paciente, foi utilizada a abordagem de equações de estimação generalizadas (GEE – *generalized estimating equations* ). Como fatores do estudo, analisamos: apresentação clínica, internação hospitalar, infecção oportunista e gestação (no caso das pacientes mulheres). Variáveis sociodemográficas e de comportamento (idade jovem, ensino médio, abuso de álcool, cocaína e crack) foram utilizadas como controle nos modelos.

Os modelos foram estratificados por sexo, por conta da presença da variável “gestação”, incluída apenas na análise das mulheres. Para ambos os sexos, foram construídos três modelos que buscaram estimar as razões de chances (RC) de adesão ao tratamento antirretroviral. O primeiro modelo considera apenas as exposições em estudo; o segundo inclui variáveis sociodemográficas de controle; e o terceiro acrescenta as variáveis de uso de drogas.

As análises foram executadas no software *Statistical Package for the Social Sciences* (SPSS), versão 18, utilizando o nível de significância de 5%. O projeto foi aprovado pelo Comitê de Ética em Pesquisa da Universidade Federal do Rio Grande do Sul (CEP/UFRGS) e os pesquisadores assinaram um termo de compromisso de uso dos dados.

## RESULTADOS

As características sociodemográficas e comportamentais dos pacientes são apresentadas na Tabela 1 . Dos 528 pacientes, 270 (51,1%) são homens e 258 (48,9%) são mulheres, com idade mediana de 37 anos (IQR = 30–46) e 35 anos (IQR = 27–44), respectivamente.

**Tabela 1:** Características sociodemográficas e comportamentais dos pacientes, por sexo – mediana (IQR) ou n (%).

Variável	Homens (n = 270)	Mulheres (n = 258)	p
Idade - Mediana (IQR)	37 (30–46)	35 (27–44)	0,073^a^
Escolaridade (anos)			
≤ 4	39 (14,4%)	42 (16,3%)	0,411^b^
5–8	137 (50,8%)	140 (54,3%)	
≥ 8	94 (34,8%)	76 (29,4%)	
Situação conjugal			0,367^b^
Solteiro	139 (51,9%)	116 (45,7%)	
Casado/morando junto	78 (29,1%)	83 (32,7%)	
Namorando	51 (19,0%)	55 (21,7%)	
Categoria de exposição ao HIV			< 0,001^c^
Heterossexual	105 (38,9%)	219 (84,9%)	
Homossexual	48 (17,8%)	2 (0,8%)	
Uso de drogas injetáveis	10 (3,7%)	0 (0%)	
Transmissão vertical	0 (0%)	0 (0%)	
Ignorado	107 (39,6%)	37 (14,3%)	
Histórico de abuso de álcool?			< 0,001^b^
Sim	88 (32,6%)	32 (12,4%)	
Não	182 (67,4%)	226 (87,6%)	
Histórico de uso de cocaína?			0,003^b^
Sim	61 (22,6%)	33 (12,8%)	
Não	209 (77,4%)	225 (87,2%)	
Histórico de uso de crack?			0,167^b^
Sim	39 (14,4%)	27 (10,5%)	
Não	231 (85,6%)	231 (89,5%)	

^a^Teste t de Student.

^b^Teste de igualdade de proporções baseado na estatística qui-quadrado de Pearson.

^c^Teste exato de Fisher.

Analisando o uso de drogas lícitas e ilícitas, o histórico de uso de homens é maior do que o de mulheres: 32,6% dos homens e 12,4% das mulheres possuem histórico de abuso de álcool (p < 0,001); 22,6% dos homens e 12,8% das mulheres apresentam histórico de cocaína (p = 0,003), e 14,4% dos homens possuem histórico de uso de crack, enquanto nas mulheres a proporção é de 10,5% (p = 0,167).

Foi expressivo o número de prontuários analisados que não continham informações referentes à categoria de exposição ao HIV dos pacientes: 39,6% entre os homens e 14,3% entre as mulheres. Cerca de 84,9% das mulheres da amostra adquiriram o HIV por meio de relações heterossexuais – o que representa quase a totalidade das pacientes mulheres com a informação disponível. Nos homens, além da grande proporção de prontuários sem informação, 38,9% dos pacientes relataram infecção através de relações heterossexuais; 17,8% através de relações homossexuais e 3,7% devido ao uso de drogas injetáveis (p < 0,001).

Quanto à situação conjugal, as proporções se apresentaram semelhantes entre homens e mulheres: 51,9% deles e 45,7% delas eram solteiros(as), enquanto 29,1% dos homens e 32,7% das mulheres eram casadas(os) ou moravam junto com o parceiro. Já a variável escolaridade mostra que a maioria dos participantes da amostra possuem de 5 a 8 anos de estudo e que a proporção de pessoas com até 4 anos de estudo foi de 14,4% para os homens e 16,3% para as mulheres. As variáveis escolaridade e situação conjugal não apresentaram diferença estatisticamente significativa entre os grupos.

A Tabela 2 descreve as consultas dos pacientes em relação à ocorrência de falhas na adesão, apresentação clínica, internação hospitalar, infecções oportunistas e gestação (n = 3.429). Referente à adesão ao TARV, os homens relataram aderência em 86,1% das consultas, enquanto as mulheres, em 83,9%. A apresentação clínica dos pacientes também foi semelhante entre os dois grupos: em 82,4% das consultas dos homens e 81,5% das consultas das mulheres não foram relatados sintomas clínicos. Os homens e mulheres passaram por internações hospitalares por conta de complicações relacionadas ao HIV em 2,2% e 1,9% das consultas, respectivamente. Infecções oportunistas ocorreram em 10 consultas (0,6%) para os homens, e 4 (0,2%) consultas para as mulheres. Não foram encontradas diferenças estatisticamente significativas entre as consultas dos dois grupos. Por fim, verificou-se que as pacientes mulheres estavam em gestação em 9,3% de todas as consultas.

**Tabela 2:** Fatores associados ao número de consultas em homens e mulheres – n (%).

Fator	Número de consultas		p^a^

	Homens (n = 1.663)	Mulheres (n = 1.766)	
Esteve em adesão regular desde a consulta anterior?			0,285
Sim	1.432 (86,1%)	1.482 (83,9%)	
Não	231 (13,9%)	284 (16,1%)	
Apresentação clínica na consulta			0,534
Sintomático	293 (17,6%)	327 (18,5%)	
Assintomático	1370 (82,4%)	1.439 (81,5%)	
Ocorrência de internação hospitalar?			0,773
Sim	36 (2,2%)	33 (1,9%)	
Não	1627 (97,8%)	1.733 (98,1%)	
Ocorrência de infecção oportunista?			0,316
Sim	10 (0,6%)	4 (0,2%)	
Não	1653 (99,4%)	1.762 (99,8%)	
Em gestação durante a consulta?			
Sim	-	165 (9,3%)	
Não	-	1.601 (90,7%)	

^a^Estimado por meio do modelo de regressão logística via equações de estimação generalizadas.

A Tabela 3 apresenta estimativas de razão de chances de adesão ao TARV para os homens. No modelo 1, verificou-se um aumento nas chances de adesão à terapia antirretroviral nas consultas em que os pacientes estavam assintomáticos (RC = 1,46; IC95% 1,09–1,97) e não foram hospitalizados por conta de complicações do HIV (RC = 1,43; IC95% 1,06–1,92). A variável “infecção oportunista” não se mostrou estatisticamente significativa.

**Tabela 3:** Estimativas da razão de chances de adesão à terapia antirretroviral em homens.

Fatores	Razão de chances (IC95%)		

	Modelo 1	Modelo 2	Modelo 3
Apresentação clínica			
Sintomático	1	1	1
Assintomático	1,46^a^(1.09–1,97)	1,43^a^(1,06–1,92)	1,43^a^(1,05–1,93)
Internação hospitalar			
Sim	1	1	1
Não	1,86^a^(1,00–3,46)	1,85 (0,99–3,45)	1,82 (0,96–3,44)
Infecção oportunista			
Não	1	1	1
Sim	0,69 (0,19–2,50)	0,70 (0,19–2,51)	0,65 (0,16–2,54)
Idade (anos)			
8–24	-	1	1
≥ 24	-	0,68 (0,27–1,72)	0,62 (0,24–1,58)
Escolaridade (anos)			
< 8	-	1	1
> 8	-	2,32^a^(1,27–4,23)	1,82 (0,97–3,41)
Histórico de abuso de álcool			
Sim	-	-	1
Não	-	-	1,42 (0,81–2,47)
Histórico de uso de cocaína			
Sim	-	-	1
Não	-	-	0,85 (0,45–1,60)
Histórico de uso de crack			
Sim	-	-	1
Não	-	-	2,35^a^(1,20–4,57)

^a^p < 0,05.

No modelo 2, com variáveis sociodemográficas, estar assintomático (RC = 1,43; IC95% 1,06–1,92) e sem registro de hospitalização (RC = 1,85; IC95% 0,99–3,45) permaneceram aumentando as chances de adesão dos homens, embora a segunda variável tenha perdido significância estatística. Neste modelo, pacientes com mais de 8 anos de estudo têm aumento nas chances de adesão (RC = 2,32; IC95% 1,27–4,23). Já no modelo 3, somente apresentação clínica permaneceu significativa, aumentando as chances de adesão em cerca de 43% na ausência de sintomas (RC = 1,43; IC95% 1,05–1,93). Com relação ao uso de drogas, nunca ter usado crack aparece como fator associado à aderência (RC = 2,35; IC95% 1,20–4,57).

A Tabela 4 apresenta os três modelos apenas com as consultas das mulheres. No primeiro, estar em gestação mais do que dobra as chances de aderir ao tratamento (RC = 2,71; IC95% 1,49–4,91), enquanto a ausência de sintomas aumenta as chances, embora sem significância estatística.

**Tabela 4:** Estimativas da razão de chances de adesão à terapia antirretroviral para mulheres.

Fatores	Razão de chances (IC95%)		

	Modelo 1	Modelo 2	Modelo 3
Apresentação clínica			
Sintomático	1	1	1
Assintomático	1,18 (0,89–1,57)	1,20 (0,90–1,60)	1,22 (0,91–1,64)
Internação hospitalar			
Sim	1	1	1
Não	0,84 (0,37–1,93)	0,83 (0,36–1,91)	0,83 (0,36–1,92)
Infecção oportunista			
Não	1	1	1
Sim	2,15 (0,12–38,15)	2,19 (0,10–45,88)	2,23 (0,17–28,62)
Gestação			
Não	1	1	1
Sim	2,71^a^(1,49–4,91)	3,07^a^(1,72–5,48)	3,28^a^(1,83–5,89)
Idade (anos)			
18–24	-	1	1
≥ 24	-	1,79^a^(1,08–2,96)	1,82^a^(1,09–3,02)
Escolaridade (anos)			
< 8	-	1	1
> 8	-	1,29 (0,78–2,13)	1,22 (0,74–2,03)
Histórico de abuso de álcool			
Sim	-	-	1
Não	-	-	0,63 (0,29–1,34)
Histórico de uso de cocaína			
Sim	-	-	1
Não	-	-	2,54^a^(1,32–4,88)
Histórico de uso de crack			
Sim	-	-	1
Não	-	-	1,06 (0,50–2,22)

^a^p < 0,05.

Adicionando as variáveis sociodemográficas, o modelo 2 permanece com resultados semelhantes ao anterior, no qual a gestação (RC = 3,07; IC95% 1,72–5,48) aumenta as chances de aderência em quase três vezes. A variável idade também apresenta associação, aumentando as chances em pessoas acima de 24 anos (RC = 1,79; IC95% 1,08–2,96). Por último, no modelo 3, as pacientes em gestação na consulta apresentam mais que o triplo de chance de adesão (RC = 3,286; IC95% 1,832–5,895), enquanto não ser jovem (RC = 1,82; IC95% 1,09–3,02), e não usar cocaína (RC = 2,54; IC95% 1,32–4,88) aumentam significativamente as chances de aderir.

## DISCUSSÃO

Diversos estudos apontam fatores sociodemográficos e de comportamento que podem influenciar nas chances dos pacientes aderirem ao TARV. Em muitos destes trabalhos, a adesão é tratada de forma “definitiva” e pouco flexível: o indivíduo é aderente, ou não é^1^. No entanto, não são apenas características sociodemográficas que podem impactar na aderência ao tratamento, uma vez que eventos pontuais que ocorrem ao longo da vida dos pacientes também podem influenciar nessas chances^11^. Entre estes eventos, destacamos: ocorrência de uma gestação; hospitalização; infecção por doença oportunista; e apresentação clínica.

Neste estudo, mulheres em gestação apresentam até 3,28 de chance de adesão ao TARV em comparação às mulheres que não estão gestantes. A literatura aponta que a gravidez pode impactar na adesão porque: (1) muitas mulheres iniciam o tratamento juntamente ao pré-natal, em que parte descobre sua sorologia para o HIV; (2) há uma atenção das políticas para essa população, focadas principalmente na atenção materno-infantil; (3) existem sentimentos e medos, por parte da gestante, de transmitir o HIV para o filho^5 , 12^.

De fato, diversos são os incentivos empregados no âmbito do pré-natal para estimular a adesão ao TARV, por exemplo: maior atenção das equipes de saúde com as gestantes; rigor no acompanhamento das pacientes para evitar transmissão vertical do HIV; busca ativa de gestantes que não comparecem aos serviços de saúde etc. Dessa maneira, é esperado que esses incentivos estimulem um aumento na adesão durante a gravidez. Em outra análise dos dados da Coorte de Alvorada, evidenciou-se que as mulheres gestantes demoram menos tempo para atingirem carga viral indetectável para o HIV se comparado com as pacientes não gestantes^7^. Em contrapartida, diversos trabalhos apontam que, após o parto, aumentam as chances de falhas na adesão ou até mesmo abandono do tratamento^5 , 13 , 14^.

Isso indica que a gestação pode ser um evento pontual que impacta positivamente na aderência ao tratamento justamente por conta da maneira como essa população é monitorada no período do pré-natal, em que recebem mais atenção das políticas de saúde e maiores estímulos para a construção do vínculo com os serviços de saúde. Após o término da gestação, no entanto, esses estímulos são enfraquecidos.

O resultado deste estudo contribui para o entendimento do efeito da gestação na vida das mulheres vivendo com HIV e é relevante principalmente analisando a incidência de diagnósticos de HIV em gestantes no país e na região metropolitana que Alvorada está inserida. No Brasil, a taxa de detecção do HIV em gestantes cresceu 38,1% nos últimos 10 anos. Entre as capitais brasileiras, Porto Alegre registrou a maior taxa de detecção de HIV em gestantes no país, em 2018^15^.

Estas análises sugerem que os homens da amostra buscam manter a correta adesão, mesmo quando não possuem complicações do HIV, diferente de outros estudos com doenças crônicas que demonstram que a ausência de sintomas faz os pacientes deixarem de usar a medicação devido à falta de compreensão a respeito do curso da doença ou por julgarem que não é mais necessário o uso dos medicamentos^22 , 23^.

Contudo, esse resultado também pode ser analisado através da relação direta entre a adesão ao TARV e a esperada melhora sintomática dos pacientes, uma vez que a efetividade do tratamento e a ausência de sintomas dependem diretamente da adesão. Estudos mostram que para garantir supressão viral e uma redução significativa no risco de morte é preciso que os pacientes tomem corretamente pelo menos 95% da medicação prescrita^20 , 21^.

Analisando a relação da apresentação clínica e da aderência na literatura, trabalhos mostram que a ausência de sintomas pode ser razão para aderir aos medicamentos, embora a melhora sintomática esteja, em alguns casos, associada ao abandono do tratamento, uma vez que o paciente pode julgar que não precise mais da medicação^1 , 11^. Por outro lado, apresentar sintomas também pode fazer com que o paciente opte por abandonar o tratamento por julgar que a piora sintomática possui relação com os medicamentos^16^.

Esse tipo de “autogestão” dos medicamentos^17^e possibilidade de escolha diária em usá-los ou não é comum em tratamentos longos como de doenças crônicas. Após o desenvolvimento da terapia antirretroviral, a aids passou por uma transformação sobre o seu aspecto de doença aguda para crônica. Isto é, se antes a doença estava associada à certeza de morte, hoje é possível viver com o vírus do HIV cotidianamente^18 , 19^. Assim como para outras doenças crônicas, a adesão regular do paciente ao tratamento pode não ocorrer mesmo com sintomas da doença. Ela também é dificultada porque, em alguns casos, pacientes não encaram o tratamento como sendo um processo longo e contínuo, característico de doenças crônicas^11^.

Entre as variáveis sociodemográficas e comportamentais, foi encontrada associação entre idade e adesão ao TARV, onde possuir mais de 24 anos aumenta a chance de aderi-lo em até 82% entre as mulheres. Diversos são os estudos que investigam fatores associados à adesão ao TARV e apontam que pessoas jovens possuem menos chance de adesão tanto para o tratamento antirretroviral como para outras doenças crônicas^1 , 3 , 8 , 9 , 22^. Além das dificuldades em aderir, dados recentes têm mostrado que jovens são um dos grupos que mais tiveram aumento nas taxas de detecção de HIV nos últimos anos, evidenciando a vulnerabilidade dessas pessoas no cenário atual da epidemia. Entre os jovens na faixa etária de 20 a 24 anos, a taxa de detecção cresceu 94,6% na última década^15^.

Já o uso de drogas como crack e cocaína é apontado na literatura diversas vezes, também, como sendo um fator de grande impacto no TARV^1^. Uma ampla revisão da literatura desenvolvida sobre o tema mostra que são relatados diversos casos em que o uso de substâncias aparece associado a complicações do HIV, uma vez que reduzem as chances de adesão dos pacientes ao tratamento antirretroviral^24^. Neste estudo, verificou-se que não possuir histórico de uso de crack e cocaína aumenta significativamente as chances de adesão. Outras pesquisas têm demonstrado que o uso de drogas tem efeito negativo sobre as chances de aderir, tanto em homens quanto mulheres e em países ricos ou pobres^25 , 26^. Trabalhos e revisões envolvendo o uso de TARV também apontam que pessoas em uso de drogas apresentam maiores dificuldades em adesão, mesmo com presença de sintomas e quadros clínicos evoluídos à aids^1 , 2 , 24^.

É preciso ressaltar que, diferente de outros trabalhos, nossa amostra foi composta por consultas, o que pode estar relacionado à alta adesão encontrada no estudo (86,1% nas consultas dos homens e 83,9% nas consultas das mulheres). Espera-se que os pacientes que compareçam às consultas estejam mais propensos a seguir o tratamento, enquanto pessoas em adesão irregular tendem a faltar mais vezes ou até interrompem o tratamento, fazendo a proporção de consultas com pacientes em adesão na amostra aumentar naturalmente. Além disso, é preciso destacar que 1.170 consultas (17,1% de todas as consultas da coorte) foram excluídas das análises porque os pacientes não compareceram à ela. Por fim, outro fator que pode estar impactando essa proporção elevada de consultas em adesão é a própria maneira como a variável foi aferida. Em alguns casos, o autorrelato dos pacientes pode ser superestimado devido à preocupação em não desapontar os profissionais de saúde que prestam o atendimento^11^.

A amostra do estudo contempla uma população em vulnerabilidade social. Mais da metade dos pacientes possuem até oito anos de estudo, o que equivale ao ensino fundamental, e aproximadamente um quarto possuem histórico de uso de crack e/ou cocaína. Alvorada está situada na Região Metropolitana de Porto Alegre e é o 2º município com menor PIB per capita do Rio Grande do Sul^28^. Além disso, o município apresenta escores menores que a média nacional em estatísticas da educação e desenvolvimento^27 , 28^.

Este estudo utilizou dados secundários, o que limita a análise dos dados somente para informações registradas nos prontuários. Nesse sentido, não foram exploradas outras características associadas à adesão que aparecem discutidas na literatura. Além disso, entre as variáveis selecionadas para as análises, em muitas consultas não foram encontradas as informações, reduzindo significativamente o número de unidades de análise da amostra. Também é importante reiterar que a maneira como a adesão foi aferida pode influenciar os resultados, uma vez que os pacientes podem referir maior aderência aos profissionais de saúde para não decepcioná-los^11^.

Por outro lado, a grande contribuição do estudo é olhar a adesão na trajetória dos pacientes. Essa perspectiva permitiu demonstrar como alguns eventos pontuais que ocorrem na trajetória de pacientes em situação de vulnerabilidade podem impactar na adesão ao tratamento. A influência da gestação sobre a aderência sugere que o pré-natal possui importância para o tratamento de gestantes vivendo com HIV. No entanto, assim como a gestação, o período do pré-natal é passageiro, e não contempla todas as mulheres.

Nesse sentido, novas estratégias que estimulem uma adesão permanente merecem ser pensadas para a garantia do sucesso terapêutico. Sobretudo, no caso das mulheres, é crucial a mudança de estratégias e implementação de políticas públicas que dão suporte às mulheres também após a gestação, quando a chance de adesão é reduzida. Além de fomentar o bem-estar das pacientes, medidas para o fortalecimento da retenção de mulheres puérperas aos serviços de saúde (como visitas domiciliares, consultas de adesão e efetiva vinculação com à atenção básica) precisam ser estimuladas, também, para o controle do risco de transmissão vertical por aleitamento, muitas vezes negligenciado pelas políticas de saúde.

Os resultados do trabalho apontam que uma boa apresentação clínica aumenta as chances de adesão ao TARV para homens da amostra. Neste sentido, nossos dados indicam a importância da integração das estratégias de prevenção e assistência ao HIV, visto que pacientes com boa adesão tendem a ter carga viral indetectável, diminuindo significativamente as chances de transmissão do vírus.
